# PCB11 Metabolite, 3,3’-Dichlorobiphenyl-4-ol, Exposure Alters the Expression of Genes Governing Fatty Acid Metabolism in the Absence of Functional Sirtuin 3: Examining the Contribution of MnSOD

**DOI:** 10.3390/antiox7090121

**Published:** 2018-09-15

**Authors:** Sinthia Alam, Gwendolyn S. Carter, Kimberly J. Krager, Xueshu Li, Hans-Joachim Lehmler, Nukhet Aykin-Burns

**Affiliations:** 1 Division of Radiation Health, Department of Pharmaceutical Sciences, College of Pharmacy, University of Arkansas for Medical Sciences, Little Rock, AR 72205, USA; SAlam2@uams.edu (S.A.); Gwendolyn.Carter@np.edu (G.S.C.); KJKrager@uams.edu (K.J.K.); 2 Department of Occupational and Environmental Health, College of Public Health, The University of Iowa, Iowa City, IA 52242, USA; xueshu-li@uiowa.edu (X.L.); hans-joachim-lehmler@uiowa.edu (H.-J.L.)

**Keywords:** Sirtuin 3, MnSOD, PCB 11, 4OH-PCB11, fatty acid metabolism, mitochondria

## Abstract

Although the production of polychlorinated biphenyls (PCBs) is prohibited, the inadvertent production of certain lower-chlorinated PCB congeners still threatens human health. We and others have identified 3,3’-dichlorobiphenyl (PCB11) and its metabolite, 3,3’-dichlorobiphenyl-4-ol (4OH-PCB11), in human blood, and there is a correlation between exposure to this metabolite and mitochondrial oxidative stress in mammalian cells. Here, we evaluated the downstream effects of 4OH-PCB11 on mitochondrial metabolism and function in the presence and absence of functional Sirtuin 3 (SIRT3), a mitochondrial fidelity protein that protects redox homeostasis. A 24 h exposure to 3 μM 4OH-PCB11 significantly decreased the cellular growth and mitochondrial membrane potential of SIRT3-knockout mouse embryonic fibroblasts (MEFs). Only wild-type cells demonstrated an increase in Manganese superoxide dismutase (MnSOD) activity in response to 4OH-PCB11–induced oxidative injury. This suggests the presence of a SIRT3-mediated post-translational modification to MnSOD, which was impaired in SIRT3-knockout MEFs, which counters the PCB insult. We found that 4OH-PCB11 increased mitochondrial respiration and endogenous fatty-acid oxidation-associated oxygen consumption in SIRT3-knockout MEFs; this appeared to occur because the cells exhausted their reserve respiratory capacity. To determine whether these changes in mitochondrial respiration were accompanied by similar changes in the regulation of fatty acid metabolism, we performed quantitative real-time polymerase chain reaction (qRT-PCR) after a 24 h treatment with 4OH-PCB11. In SIRT3-knockout MEFs, 4OH-PCB11 significantly increased the expression of ten genes controlling fatty acid biosynthesis, metabolism, and transport. When we overexpressed MnSOD in these cells, the expression of six of these genes returned to the baseline level, suggesting that the protective role of SIRT3 against 4OH-PCB11 is partially governed by MnSOD activity.

## 1. Introduction

Polychlorinated biphenyls (PCBs) are synthetic organic chemicals consisting of a biphenyl and various numbers of chlorine atoms. For more than 50 years, PCBs were produced commercially (Aroclors) and marketed for various industrial applications, including use in capacitors, transformers, plasticizers, surface coatings, inks, adhesives, and pesticides—until their adverse effects on health became evident [1]. Chronic exposure to mixtures of PCBs is associated with a wide range of toxic effects, including hepatotoxicity, carcinogenicity, hormonal disruption, and neurotoxicity [2]. Although the production of PCBs was banned in North America in 1977, and PCBs are no longer used in manufacturing, a unique PCB congener (3,3′-dichlorobiphenyl [PCB11]) was detected in soil and air samples from New York, Chicago, and Philadelphia; notably, this congener was not originally a component of Aroclors [3,4]. The same group also identified PCB11 and other PCB congeners (due to unintended production during yellow pigment manufacturing) in commercial paints, inks, textiles, paper, cosmetics, leather, plastics, and food. Thus, the general population is exposed on a daily basis to PCBs [5]. Furthermore, Marek et al., as well as Sethi et al., detected PCB11 in human samples [6,7], and our group reported measurable levels of hydroxylated PCB11 metabolites, including 4OH-PCB11, in human blood [8]. Thus, understanding the PCB-induced health effects at a molecular level could help establish strategies to overcome the health effects of PCB exposure.

Studies from the past ten years strongly suggest that PCBs and PCB metabolites, such as 4-chlorobenzoquinone and 4OH-PCB11, induce mitochondrial reactive oxygen species (ROS) and oxidative stress in cultured cells and mice [8,9,10,11,12]. In Zhu et al. (2009), we showed that exposure to PCBs and PCB metabolites increased the activity of MnSOD, the major mitochondrial enzyme responsible for removing O_2_•^–^ [12]. Interestingly, this increase in MnSOD activity was not accompanied by an increase in MnSOD immunoreactive protein, suggesting that MnSOD was regulated post-translationally. Further, SIRT3, as the major deacetylase in mitochondria, is capable of affecting MnSOD by deacetylating its critical lysines to regulate its activity [13,14,15]. SIRT3 appears to decrease in abundance during aging as a result of a high fat-diet or metal toxicity, and in metabolic disease states such as type II diabetes [16,17,18,19,20]. Because SIRT3 regulates mitochondrial metabolism during stress, it has the potential to be an important player in PCB-induced changes to mitochondrial function and metabolism.

Here, we conducted a series of experiments to determine the effects of 4OH-PCB11 on mitochondrial function and metabolism in the presence and absence of functional SIRT3 in vitro. By overexpressing MnSOD, we also investigated the role of this mitochondrial enzyme in the 4OH-PCB11-induced metabolic changes.

## 2. Materials and Methods 

### 2.1. Synthesis and Authentication of 4OH-PCB11

4-OH PCB 11 was synthesized analogously from 3-chloro-4-methoxybromobenzene and 3-chloroboronic benzene using a modified Suzuki-coupling reaction with Pd_2_(dba)_3_ /2-dicyclohexylphosphino-2′,6′-dimethoxybiphenyl (DPDB) as the catalyst [21], followed by deprotection of the methoxylated intermediate with boron tribromide (BBr_3_) [22]. 4OH-PCB11 was obtained as an off-white power with a purity of 99.6%, based on relative peak area determined with a published GC-MS method [23]. Melting point: 53–54 °C. 1H NMR (CDCl_3_, 400M Hz) δ 7.52 (d, J = 2.1 Hz, 1H), 7.50 (s, 1H), 7.39–7.26 (m, 4H), 7.09 (d, J = 8.4 Hz, 1H), 5.63 (broad s, 1H). 13C NMR (CDCl_3_, 100 M Hz) δ 151.1, 141.3, 134.7, 133.4, 130.0, 127.4, 127.2, 127.1, 126.8, 124.8, 120.4, 116.6. GC-MS (EI, 70 eV, *m/z*, %): 237.9 ([M]+, 95), 168.0 (14), 138.9 (100), 113.0 (11), 87.0 (14), 63 (16). The gas chromatogram and mass spectrum of 4OH-PCB 11 are shown in Figure S1.

### 2.2. Mouse Embryonic Fibroblast Isolation and Culture Conditions

SIRT3 wild-type (WT) and knockout (KO) mouse embryonic fibroblasts (MEFs) from the B6/Sv129 background were isolated [13]. Mouse embryos were dissected on day 13 post coitum into sterile Dulbecco’s phosphate buffered saline. The embryos were transferred to a clean tissue-culture dish containing trypsin/EDTA solution and dissociated by aspirating through a sterile needle. The dissociated tissue was then incubated for 5 min at 37 °C. Trituration and incubation were repeated three times. The contents were then transferred to a tube containing an equal volume of Dulbecco’s Modified Eagle Medium (DMEM) containing L-glutamine and sodium pyruvate and supplemented with 10% fetal bovine serum, 4-(2-hydroxyethyl)-1-piperazineethanesulfonic acid (HEPES), non-essential amino acids, and 1% penicillin/streptomycin. The tubes were allowed to stand for 3 to 5 min at room temperature, and the solution was carefully separated and placed into a new conical tube, avoiding large tissue pieces. The solution was centrifuged for 5 min at 200× *g* at room temperature. After discarding the supernatant, the pellet was resuspended in 10 mL fresh DMEM medium with penicillin/streptomycin. The cells were plated on tissue culture plates and grown until confluent (2 to 5 days); the cultures were maintained at 37 °C, 4% O_2_, and 5% CO_2_, and all experiments were performed using cells between passages two and six.

### 2.3. Doubling Time

MEFs were plated in 60-mm culture dishes. Cells were counted with a Countess Automatic Cell Counter (Invitrogen, Thermo Fisher Scientific, Carlsbad, CA, USA) for five days in the presence and absence of 3 μM 4OH-PCB11, which was synthesized and characterized by the University of Iowa Superfund Research Program Synthesis Core [8]. The doubling time was calculated from the exponential part of the growth curve with the equation: Doubling Time = 0.693 × t/ln(Nt/N0), where T = time, Nt = number of cells at time t, and N0 = number of cells at the initial time.

### 2.4. Western Blot Analysis

MEFs were cultured in complete MEF medium overnight and then treated for 24 h with 3 μM 4OH-PCB11 or vehicle (dimethyl sulfoxide, DMSO, 0.1% *v/v*). Cells were collected, washed with cold phosphate buffered saline (PBS), and lysed in a radioimmunoprecipitation assay (RIPA) buffer with protease inhibitors (Roche, Indianapolis, IN, USA). Protein concentrations were determined with the Pierce bicinchoninic acid (BCA) Protein Assay Kit (Thermo Fisher Scientific). Protein samples were diluted in 1X Laemmli Sample buffer with 5% 2-beta mercaptoethanol (2-BME) in order to load 30 μg of protein to each lane of a 5–14% gradient Mini-PROTEAN TGX gel (Bio-Rad, Hercules, CA, USA). Proteins were transferred to a nitrocellulose membrane and blocked for 1 h in Odyssey Blocking Buffer (LI-COR, Lincoln, NE, USA), then incubated overnight in a 1:1000 dilution of the target antibody (MnSOD, Millipore, Burlington, MA, USA; SIRT3, Cell Signaling, Danvers, MA, USA). β-actin was used as a loading control (Cell Signaling). After washing, the blots were incubated in a 1:10,000 dilution of LI-COR anti-mouse/anti-rabbit fluorescent antibodies for 40 min and visualized with the Odyssey Fc Imaging System (LI-COR).

### 2.5. Mitosox and JC-1 Labeling for Mitochondrial ROS and Mitochondrial Membrane Potential

MEFs were incubated in complete MEF medium overnight and then treated for 24 h with 3 μM 4OH-PCB11 or vehicle (DMSO, 0.1% *v/v*). Mitochondrial reactive oxygen species (ROS) and mitochondrial membrane potential were measured with Mitosox (2 μM for 15 min at 37 °C, Invitrogen, Thermo Fisher Scientific) and JC-1 labeling, respectively (5 μ/mL, 30 min at 37 °C Invitrogen, Thermo Fisher Scientific). The cells were then washed, placed on ice, filtered, and analyzed by flow cytometry (λex = 405 nm and λem = 585/42 nm bandpass filter [Mitosox]; λex = 488 nm and Green, λem = 530/30 nm, and Red, λem = 585/42 nm, bandpass filters for JC-1 staining). The mean fluorescence intensity (MFI) of 10,000 cells was analyzed and corrected for autofluorescence from unlabeled cells. The MFI data were presented in arbitrary units for Mitosox, and the ratio of red/green fluorescence was plotted for JC-1 [12,24].

### 2.6. MnSOD Activity and Adenoviral Transduction of MnSOD

MnSOD activity was measured based on the protocol by Spitz and Oberley. Activity was determined by measuring the rate of reduction of nitroblue tetrazolium (NBT) by the superoxide generated by xanthine oxidase via competitive inhibition by superoxide dismutase. One unit of SOD is considered the amount required to inhibit 50% of the NBT reduction. This is determined with increasing concentrations of sample to calculate the percent-inhibition curve and determine the concentration at 50% inhibition. MnSOD activity is specifically determined by adding sodium cyanide to inhibit CuZnSOD activity [25,26].

MnSOD was overexpressed in MEFs adenovirally. Replication-incompetent adenoviral vectors, AdCMV Bgl II (AdBgl II) and AdCMV MnSOD (AdMnSOD), were purchased from Viraquest (North Liberty, Iowa). Cells were plated the day before transduction. Viral particles were added to 2 mL of medium for an MOI of 100 per 60-mm dish. Cells and viral particles were incubated for 24 h, the medium was replaced, and the mixture was incubated for an additional 24 h before each experiment [25].

### 2.7. Cellular Bioenergetics Analysis and Fatty Acid Oxidation Assessment via Cellular Respirometry

Asynchronous cultures of SIRT3 WT and KO MEFs were trypsinized and plated in Seahorse XF96 cell culture plates (Agilent, Lexington, MA, USA ). The next day, cells were treated with 3 μM 4OH-PCB11 or vehicle for an additional 24 h. The cells were washed in buffered DMEM, and the medium was replaced with assay medium. Oxygen consumption rate (OCR) measurements were made using a 3-min mix, 4-min measure protocol. The parameters were determined by measuring the OCR after sequential injections of oligomycin (inhibits ATP synthase), carbonyl cyanide p-(trifluoromethoxy)phenylhydrazone (FCCP; uncouples mitochondrial inner membrane), and an antimycin A–rotenone mixture (inhibits electron transport chain (ETC) complexes III and I). OCR values were normalized to cell number and reported as pmol O_2_ consumed per min per 10,000 cells [27,28,29,30].

For fatty acid oxidation (FAO) assessment via cellular respirometry, asynchronous cultures of SIRT3 WT and KO MEFs were trypsinized and plated in Seahorse XF96 cell culture plates (15,000 cells/well, Agilent). After 24 h, cells were treated with 3 μM 4OH-PCB11 or vehicle for an additional 24 h. At 8 h before metabolic flux analysis, the culture medium was replaced with substrate-limited medium (DMEM with 0.5 mM glucose, 1.0 mM glutamine, 0.5 mM carnitine, and 1% FBS). Forty-five minutes before the beginning of oxygen consumption measurements, the media in the wells were replaced with FAO assay medium (111 mM NaCl, 4.7 mM KCl, 2.0 mM MgSO_4_, 1.2 mM Na_2_HPO_4_, 2.5 mM glucose, 0.5 mM carnitine, and 5 mM HEPES). Immediately before placing the plates into the XF analyzer, BSA or palmitate-BSA was added to distinguish between the endogenous and exogenous FAO-driven respiration. After the baseline OCR was stabilized in FAO assay medium, etomoxir (a carnitine palmitoyl transferase-1 inhibitor, 40 μM) was injected; the FAO-associated OCR was calculated as the difference in OCR before and after etomoxir injection [31].

### 2.8. Quantitative RT-PCR Analysis

Asynchronous cultures of WT and SIRT3-KO MEFs were trypsinized and plated. After 24 h, cells were treated in triplicate with 0.1, 1, or 3 μM 4OH-PCB11 for an additional 24 h. To quantify mRNA expression, total RNA was extracted from MEFs with the QIAGEN RNA Extraction kit. Total RNA was reverse-transcribed with the High Capacity cDNA Reverse Transcriptase kit (Applied Biosystems, Foster City, CA, USA). qRT-PCR was performed with the Applied Biosystems SYBR Select Master mix and mouse fatty acid metabolism RT^²^ Profiler™ PCR Array (QIAGEN, Germantown, MA, USA), see Table S1, on an Applied Biosystems ViiA 7. Changes in expression were confirmed with a new set of samples and single-gene qRT-PCR, see Table S2. The cycle threshold (CT) values for the target genes were normalized to the 18S rRNA transcript. The fold difference (relative abundance) was calculated using the formula 2-ΔΔCT (changes between the CT differences of the treated and control samples), and mean values were plotted.

### 2.9. Statistical Analysis

Statistical analyses were performed with GraphPad Prism 6.0 (GraphPad Software, San Diego, CA, USA). Data are expressed as the mean ±  1SD, unless otherwise specified. One-way analysis of variance (ANOVA) with Tukey’s post-hoc analysis was used to study the differences between group means. Significance was defined as *p* < 0.05.

## 3. Results

### 3.1. Effect of 4OH-PCB11 on the Growth of WT and SIRT3-KO MEFs

**Figure 1:**
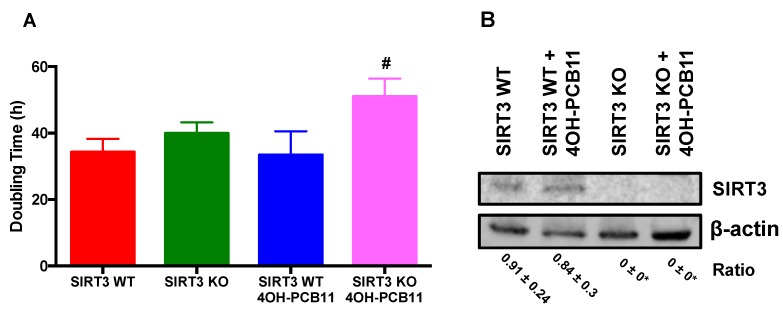
Treatment with 3 μM 4OH-PCB11 for 24 h decreased the cell growth in SIRT3-KO MEFs. (**A**) Doubling times of WT and SIRT3-KO MEFs treated with Vehicle (0.1% *v/v* DMSO) or 3 μM 4OH-PCB11 for 24 h. Data are mean doubling times of cells from six treatment dishes from two separate experiments performed in triplicate (± 1SD), *n* = 6. # *p* < 0.05, as compared to the vehicle-treated SIRT3-KO group. (**B**) Western blot analysis of Sirt3 immunoreactive protein in WT and SIRT3-KO MEFs following 24 h treatment with 3 μM 4OH-PCB11 or vehicle. β-actin was used as a loading control. The quantitation was presented as the average + 1SD ratio of SIRT3 to β-actin in fluorescence units from three different blots * *p* < 0.05 as compared to vehicle-treated SIRT3-WT group.

To establish the biological effects of PCBs on cell growth, we incubated WT and SIRT3-KO MEFs with 3 μM 4OH-PCB11 or vehicle (DMSO; 0.1% *v/v*) for 24 h. This dose was chosen based on our previous studies and is representative of the upper limit of doses measured in people living in PCB-contaminated areas [8,12]. Following treatment, cells were trypsinized then counted for five days. Doubling times (Td) were calculated as: Td = 0.693t/ln (Nt/N0), where Nt and N0 represent cell numbers at time t and zero, respectively. We found that 4OH-PCB11 significantly decreased the growth of SIRT3-KO MEFs, as indicated by an increase in doubling time; whereas, no change in doubling time was observed for WT cells, see Figure 1A. PCB treatment (24 h) did not significantly affect the viability of the cells (data not shown). It also did not alter the levels of SIRT3 in WT MEFs, and (as expected) no SIRT3 immunoreactive protein was detected in the vehicle- or 4OH-PCB11-treated KO MEFs, as shown in Figure 1B.

### 3.2. Effect of 4OH-PCB11 on Mitosox Oxidation and Mitochondrial Membrane Potential

**Figure 2:**
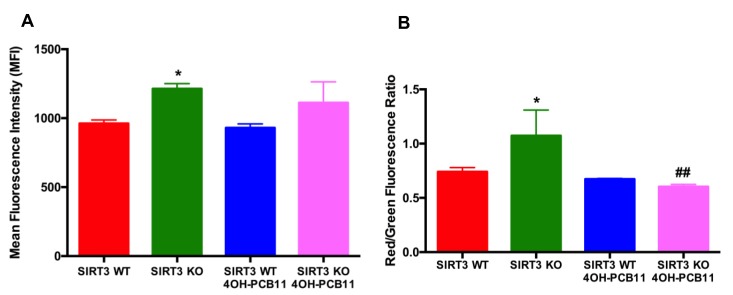
Steady-state levels of reactive oxygen species (ROS) and mitochondrial membrane potential were determined by Mitosox oxidation. (**A**) Asynchronous cultures of MEFs were incubated with 3 μM 4OH-PCB11 for 24 h. Cells were trypsinized, washed once with PBS, and labeled with 2 μM Mitosox (in 0.1% DMSO, 20 min) in PBS containing 5 mM pyruvate at 37 °C. (**B**) Mitochondrial membrane potential as measured by JC-1 (5 μg/mL, 15 min). The mean fluorescence intensity (MFI) of 10,000 cells was analyzed by flow cytometry. Samples were assayed in triplicate; data are the means ± 1SEM of two independent experiments, each containing three treatment dishes, *n* = 6. * *p* < 0.05 as compared to vehicle-treated WT control group. ## *p* < 0.01 as compared to vehicle-treated SIRT3-KO control group.

PCBs and their metabolites can affect mitochondrial superoxide levels [8,12]; thus, we used MitoSOX Red labeling to determine if 4OH-PCB11 altered the steady-state levels of superoxide originating from mitochondria. We found a marginal but statistically significant increase in MitoSOX oxidation in SIRT3-KO cells compared to WT, see Figure 2A. However, there was no change in MitoSOX oxidation after the addition of 3 μM 4OH-PCB11 to WT or KO cells for 24 h, suggesting that 4OH-PCB11 did not alter the steady-state levels of mitochondrial superoxide, see Figure 2A. To further investigate the effects of 4OH-PCB11 on mitochondria, we labeled the cells with the monomeric cationic dye JC-1. This dye fluoresces green until it enters mitochondria, where it aggregates and fluoresces red; the ratio of red/green fluorescence is used to estimate mitochondrial membrane potential. We found that under basal conditions, SIRT3-KO cells have greater mitochondrial potential than WT MEFs. There were no changes in mitochondrial membrane potential of WT MEFs when exposed to 4OH-PCB11. However, exposure to 3 μM 4OH-PCB11 for 24 h significantly decreased the mitochondrial membrane potential in SIRT3-KO cells, as shown in Figure 2B, suggesting a protective function of SIRT3 for preventing mitochondrial uncoupling during PCB exposures.

### 3.3. Effect of 4OH-PCB11 on MnSOD Abundance and Enzymatic Activity

**Figure 3:**
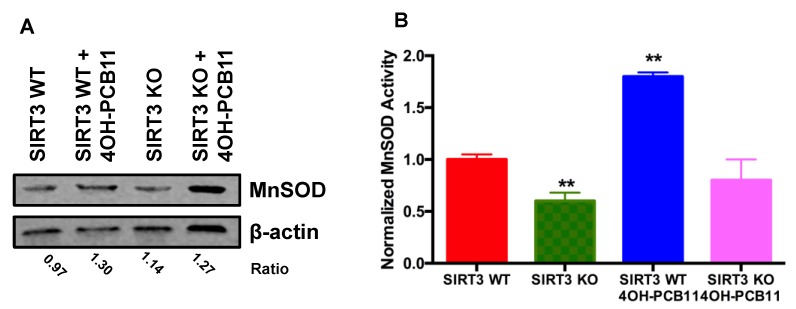
MnSOD activity was induced only in 4OH-PCB11-treated WT MEFs. (**A**) Asynchronous cultures of WT and SIRT3-KO MEFs were incubated with 3 μM 4OH-PCB11 for 24 h. Cells were harvested, scraped, and pelleted in PBS and lysed in radioimmunoprecipitation assay (RIPA) buffer for western blot. Quantitation was presented as the average ratio of MnSOD to β-actin in fluorescence units obtained from two different blots (**B**) To measure MnSOD activity, whole cell homogenates were prepared from the pellets and resuspended in 50 mM potassium phosphate buffer (pH 7.8) for spectrophotometric analysis. Data were normalized from two independent experiments. Data represent the mean of each group ± 1SEM (*n* = 6). ** *p* < 0.01 as compared to vehicle-treated WT control group.

Because 4OH-PCB11 did not alter the levels of mitochondrial superoxide as expected (based on the results of our previous study [8]), we reasoned that the activity or concentration of the major mitochondrial superoxide removal mechanism, MnSOD, could have been altered by 4OH-PCB11 (3 μM for 24 h). Western blot analysis showed no significant changes in the amount of MnSOD immunoreactive protein in WT or SIRT3-KO MEFs under any condition, as shown in Figure 3A. However, there was a significant increase in MnSOD enzymatic activity in WT MEFs compared to SIRT3-KO MEFs, see Figure 3B. This suggested that in the presence of 4OH-PCB11, MnSOD underwent a post-translational modification by SIRT3, which is known to deacetylate MnSOD and increase its function. MnSOD activity was also decreased in SIRT3-KO cells compared to WT MEFs, as expected and previously demonstrated, see Figure 3B.

### 3.4. Mitochondrial Respiration Following 4OH-PCB11 Treatment in WT and SIRT3-KO MEFs

**Figure 4:**
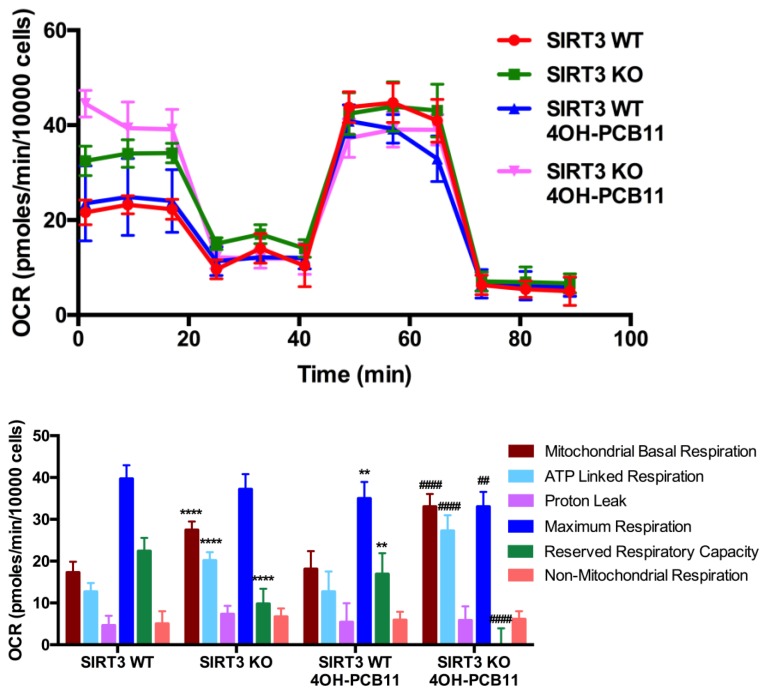
Basal and ATP-linked respiration increased in SIRT3-KO MEFs, and reserve respiratory capacity decreased. WT and SIRT3-KO MEFs were trypsinized and plated in Seahorse XF96 cell culture plates. The next day, cells were treated with 3 μM 4OH-PCB11 or vehicle for an additional 24 h. Cells were washed in buffered DMEM and then changed to assay medium. The oxygen consumption rate (OCR) was measured with sequential injections of oligomycin, FCCP, and antimycin A-rotenone mixture (upper panel). The OCR was calculated and plotted for basal, ATP-linked, proton leak, reserved respiratory, and non-mitochondrial respiration. Data shown are representative of three independent experiments. Data are the mean ± 1SEM (*n* = 18–22). ** *p* < 0.01; **** *p* < 0.0001 as compared to vehicle-treated WT control group. ## *p* < 0.01 and #### *p* < 0.0001 as compared to vehicle-treated SIRT3-KO control group.

Our finding that 4OH-PCB11 decreased the mitochondrial membrane potential in SIRT3-KO MEFs prompted us to explore how well the mitochondria function in these cells when stressed with this PCB11 metabolite. We conducted cellular respiration studies with the Seahorse XF96 Extracellular Flux Analyzer and found noteworthy differences in mitochondrial respiration between the WT and SIRT3-KO MEFs in the presence and absence of 4OH-PCB11, see Figure 4. The basal mitochondrial oxygen consumption rate (OCR) and ATP-linked respiration (determined by decrease in OCR after oligomycin injection) were significantly higher in SIRT3-KO cells than in WT cells, even WT cells in the vehicle (DMSO)-treated group. On the other hand, SIRT3-KO cells had considerably less reserve respiratory capacity (the difference between basal and maximal oxygen consumption) than WT cells. Overall, these findings demonstrate that SIRT3-KO cells possess a different profile of metabolic processes altering their mitochondrial respiration before any PCB exposure.

In the same experiment, treatment with 3 μM 4OH-PCB11 for 24 h did not alter the basal mitochondrial OCR of WT MEFs compared to DMSO-treated WT MEFs, see Figure 4. However, the same treatment significantly increased the basal mitochondrial OCR of SIRT3-KO cells compared to their DMSO-treated counterparts. ATP-linked respiration also increased in SIRT3-KO cells after 4OH-PCB11 treatment, while the reserve respiratory capacity of these cells was obliterated. There were no changes in non-mitochondrial OCR or proton leaks in any of the groups in the presence or absence of 4OH-PCB11 treatment, see Figure 4.

### 3.5. Effect of 4OH-PCB11 on Fatty Acid Oxidation-Associated Respiration in WT and SIRT3-KO MEFs

**Figure 5:**
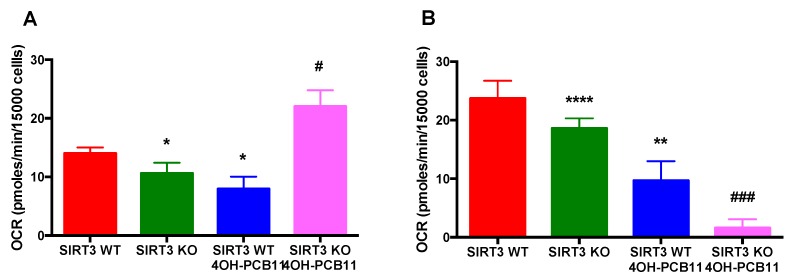
Endogenous fatty acid oxidation (FAO)-driven respiration increased only in SIRT3-KO MEFs following 4OH-PCB11 treatment. WT and SIRT3-KO MEFs were trypsinized, and 15,000 cells were plated in Seahorse XF96 cell culture plates. After 24 h, cells were treated with 3 μM 4OH-PCB11 or vehicle for an additional 24 h. At 8 h before metabolic flux analysis, the culture medium was replaced with substrate-limited medium, and 45 min before measuring the oxygen consumption rate, the medium was replaced with FAO assay medium. Immediately before placing the plates into the XF analyzer, the wells received BSA or palmitate-BSA substrate to distinguish between endogenous (**A**) and exogenous (**B**) FAO-driven respiration. After the baseline OCR was established, etomoxir (40 μM) was injected to determine the amount of FAO-associated OCR (calculated as the difference in OCR before and after etomoxir injection). Data are representative of two independent experiments. Data are the mean ± 1SEM (*n* = 18–22). * *p* < 0.05; ** *p* < 0.01; **** *p* < 0.0001 as compared to vehicle-treated WT control group. # *p* < 0.05 and ### *p* < 0.001 as compared to vehicle-treated SIRT3-KO control group.

Next, to explore the counterintuitive observation that 4OH-PCB11 decreased the growth rate and increased the OCR in SIRT3-KO MEFs, we assessed the contribution of fatty acid oxidation (FAO) to oxygen consumption. To characterize the respiration driven by FAO, we repeated our cellular bioenergetics study with the Seahorse XF96 Extracellular Flux Analyzer. The cells were plated in XF96 plates and treated with 3 μM 4OH-PCB11 for 24 h. The cells were provided with BSA or palmitate-conjugated BSA to drive FAO when other cellular substrates (glucose and glutamine) were limited and to differentiate between endogenous and exogenous FAO, respectively. FAO-associated OCR was calculated based on the OCR before and after the administration of etomoxir (carnitine palmitoyl transferase-1 inhibitor) to the wells. We found that SIRT3-KO cells had less endogenous and exogenous FAO-driven respiration than WT MEFs, see Figure 5A,B. However, treatment with 4OH-PCB11 caused an opposite response for endogenous FAO-associated OCR in WT and SIRT3-KO cells. 4OH-PCB11 significantly decreased the OCR due to endogenous FAO in WT cells, while the same treatment increased the OCR considerably in SIRT3-KO cells, suggesting fatty acid accumulation in the presence of 4OH-PCB11, providing excess endogenous substrates for FAO-linked oxygen consumption. Interestingly, when cells were treated with palmitate-conjugated BSA to initiate exogenous FAO, 4OH-PCB11 significantly decreased the OCR in both WT and SIRT3-KO MEFs, which could be due to an inhibited fatty acid transport following PCB exposure, see Figure 5B.

### 3.6. Changes in Gene Expression Associated with Fatty Acid Metabolism in WT and SIRT3-KO MEFs after 4OH-PCB11 Treatment

To determine whether the differences in endogenous and exogenous FAO-driven OCR observed for WT and SIRT3-KO cells could be contributed to FAO targets that are upstream of aerobic respiration, we performed a pathway-focused PCR to assay genes involved in regulating fatty acid metabolism. We assessed the dose–response relationship by treating WT and SIRT3-KO cells with three different concentrations of 4OH-PCB11 for 24 h (0.1, 1, or 3 μM 4OH-PCB11). Each PCR assayed 84 genes involved in fatty acid biosynthesis regulation, metabolism, and transport, as well as genes for ketone body and triacylglycerol metabolism, see Table S2.

**Figure 6:**
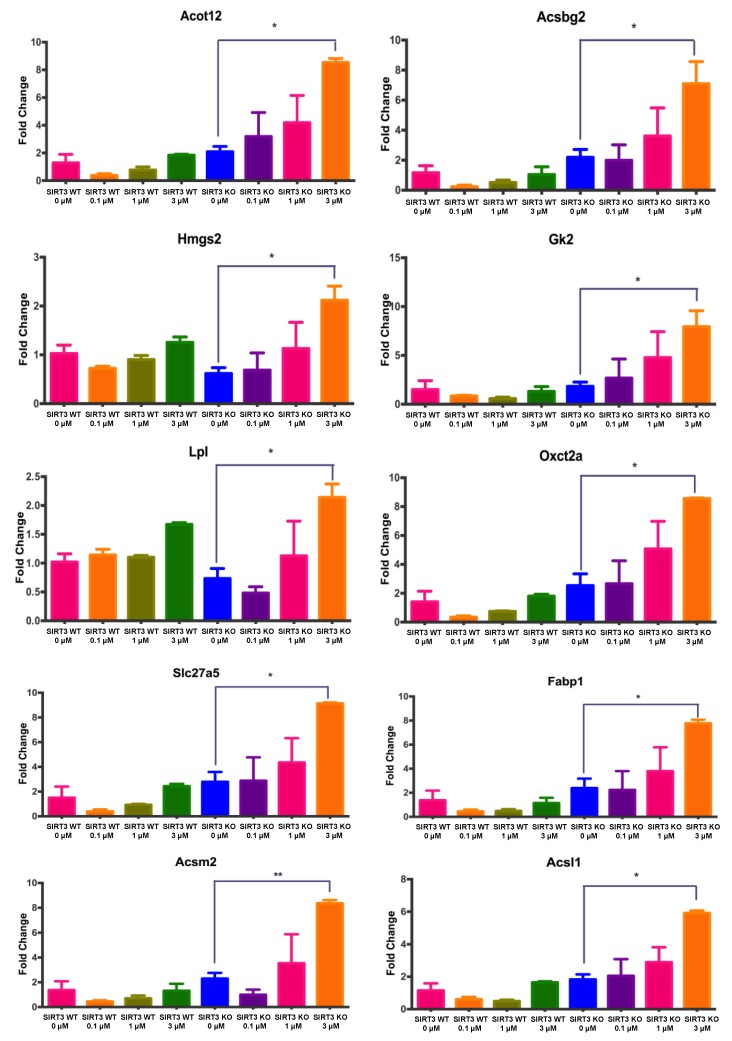
4OH-PCB11 caused increased expression of genes associated with fatty acid oxidation in SIRT3-KO MEFs. WT and SIRT3-KO MEFs were trypsinized and plated. After 24 h, cells were treated in triplicate with 0.1, 1, and 3 μM of 4OH-PCB11 for an additional 24 h. To quantitate mRNA expression for fatty acid metabolism, total RNA was extracted with a QIAGEN RNA Extraction kit. Total RNA was reverse transcribed, and qRT-PCR was performed. The CT values for the target genes were normalized to the 18S rRNA transcript, the fold difference (relative abundance) was calculated using the formula 2-ΔΔCT, and the mean was plotted. N = 3; * *p* < 0.05; ** *p* < 0.01.

We identified ten genes with at least a two-fold increase in expression and confirmed our findings with single-gene qRT-PCR on new samples: Acsbg2, Acsm2, Acsl1, Acot12, Hmgs2, Oxct2a, Gk2, Lpl, Slc27a5, and Fabp1, see Figure 6. We further observed a trend for dose-dependent increases in the expression of these genes in SIRT3-KO cells treated with 4OH-PCB11. However, only the group treated with 3 μM 4OH-PCB11 reached statistical significance, see Figure 6. There were no significant changes in gene expression in WT MEFs treated with any dose of 4OH-PCB11, as shown in Figure 6.

SIRT3 levels increase during stress which then facilitates a switch from glucose to fatty acid utilization as a source of energy via deacetylation and activation of acyl-CoA dehydrogenases. Sirt3^-/-^ mice exhibit hallmarks of fatty acid oxidation disorders such as intolerance to cold exposure during fasting [32,33]. Under stress conditions, they have lower fatty acid oxidation capacity (especially long-chain fatty acids) and lipid accumulation, suggesting impaired and inefficient fatty acid metabolism. Therefore, we propose that the expression of genes encoding for proteins regulating fatty acid metabolism is increased in SIRT3-KO cells, because the cells need increased activation of long- and medium-chain fatty acids to compensate for the low FAO end-product metabolite returns following treament with an environmental stressor, 4OH-PCB11.

**Figure 7:**
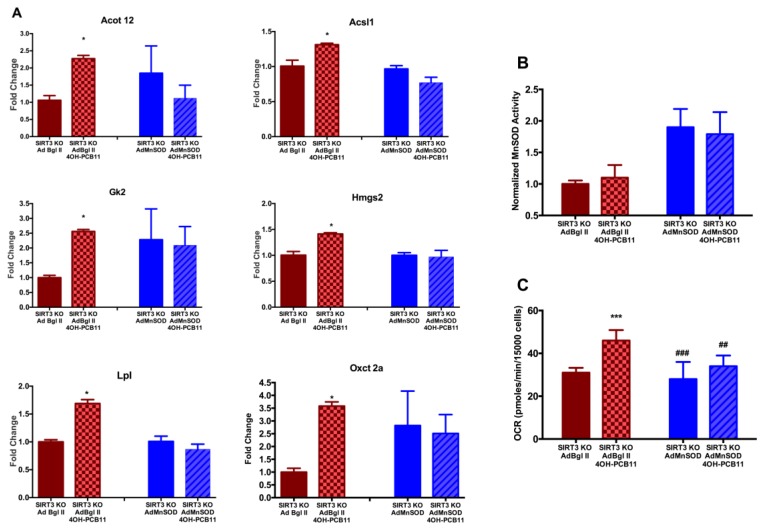
Overexpression of MnSOD could reverse the 4OH-PCB11 induced increases in expression of genes associated with FAO as well as decrease the elevated mitochondrial respiration following polychlorinated biphenyl (PCB) treatment. SIRT3-KO MEFs were incubated with replication-incompetent adenoviral vectors (100 MOI), AdCMV Bgl II (AdBgl II), or AdCMV MnSOD (AdMnSOD) for 24 h. Then, the medium was changed, and the cells were incubated for an additional day before treatment with 3 μM 4OH-PCB11 or vehicle for 24 h. qRT-PCR was repeated for the FAO pathway genes that were significantly upregulated in the previous experiment, see Figure 6 (*n* = 3). * *p* < 0.05 compared to AdBgl II control (**A**). MnSOD activity was measured in cell homogenates. Data represents the average and error range of two independent treatment dishes per group (**B**) The oxygen consumption rate (OCR) was measured with Seahorse Extracellular Flux analyzer. Data are the mean ± 1SEM of independently infected and treated 14–16 wells. *** *p* < 0.001 as compared to vehicle-treated AdBgl II group. ## *p* < 0.01 and ### *p* < 0.001 as compared to vehicle-treated AdMnSOD group (**C**).

Elevation in SIRT3 activity increases the MnSOD activity to prevent increased O_2_•^–^ generated from external stressors [14,15,34]. We also propose if we can increase MnSOD activity in SIRT3 KO cells, we can revert these alterations in the expression of FAO-related genes. To establish this causal link between increased MnSOD activity and PCB-induced changes in FAO-related gene expression, we transfected MEFs with adenoviral MnSOD. Overexpressing MnSOD in SIRT3-KO MEFs prevented the PCB-induced increase in six of our ten genes of interest: Acot12, Acsl1, Gk2, Hmgs2, Lpl, and Oxct2a, see Figure 7A. Increased enzymatic activity, as shown in Figure 7B, via adenoviral overexpression of MnSOD also decreased basal mitochondrial OCR of the 4OH-PCB11 treated SIRT3-KO MEFs to the levels of vehicle-treated cells, see Figure 7C. These results suggest that MnSOD plays a unique role in regulating mitochondrial function as well as the expression of genes involved in regulating fatty acid biosynthesis, metabolism, and transport during exposure to 4OH-PCB11.

## 4. Discussion

Cellular injury and oxidative stress induced by PCBs have been studied extensively, and the effects are widely accepted. Likewise, PCB-induced morphologic and functional changes to mitochondria are well documented [35,36,37]; the depolarization of mitochondrial membrane potential and inhibition of ETC activity following PCB exposure have been shown in vitro and in vivo [38,39,40,41]. The discovery of nonlegacy (non-Aroclor) PCB congeners, such as PCB11, in current commercial goods (e.g., paint, inks, textiles) has once again focused the attention of the scientific community on PCBs [3,5,42,43,44]. In 2013, we reported the existence of hydroxylated PCB11 metabolites, including 4OH-PCB11, in circulating human blood [8]. Because of the relatively short history of research on nonlegacy PCBs, our knowledge of their biological effects on mammalian metabolism is limited.

The mitochondrion is a major target for PCBs and PCB metabolites. We showed that PCBs and their metabolites induced oxidative stress by increasing mitochondrial superoxide (O_2_•^–^) and hydrogen peroxide (H_2_O_2_) [8,12]. Chronic exposure to PCBs and their metabolites significantly altered antioxidant enzyme profiles, including the activity of manganese superoxide dismutase (MnSOD), with no changes in protein level [8,12]. Notably, SIRT3, the primary mitochondrial NAD+-dependent deacetylase, regulates the enzymatic activity of MnSOD post-translationally in response to exogenous stress [13,14,15]. Collectively, these results strongly suggest that SIRT3 could play a significant role as an “adaptive-response protein” during PCB exposure because it can regulate mitochondrial function and oxidant detoxification processes by removing acetyl groups from lysine-modified proteins (e.g., increased MnSOD activity via deacetylation) responsible for maintaining redox homeostasis.

In the current study, we observed that the cellular effects of 4OH-PCB11 were more pronounced in SIRT3-KO MEFs than in WT. Short-term (24 h) treatment with 3 μM 4OH-PCB11 significantly increased the doubling time of SIRT3-KO cells (by decreasing cell growth), while WT cells were not affected. Similarly, this metabolite also decreased the mitochondrial membrane potential of SIRT3-KO MEFs, suggesting that 4OH-PCB11 may influence mitochondrial function and lead to alterations in oxidative metabolism. Interestingly, no cells displayed an increase in the steady-state levels of mitochondrial O_2_•^–^ following 4OH-PCB11 treatment, although we did not assess the flux of superoxide radical formation and the data only represented a single time point (24 h) immediately following 4OH-PCB11 treatment. To determine whether MnSOD, the major O_2_•^–^ detoxifying enzyme in mitochondria, responded to 4OH-PCB11, we assessed its abundance and enzymatic activity. Under basal conditions, MnSOD activity was lower in SIRT3-KO MEFs than in WT MEFs, while exhibiting similar levels of MnSOD protein, see Figure 3A. Treating WT MEFs with 3 μM 4OH-PCB11 increased MnSOD enzyme activity, but this was not accompanied by an increase in MnSOD immunoreactive protein, suggesting that PCB increased O_2_•^–^ levels to trigger a stress response by mitochondrial SIRT3, see Figure 3A,B. We reason that SIRT3 could deacetylate the critical lysines in MnSOD to increase its enzymatic activity, facilitating the removal of 4OH-PCB11-induced O_2_•^–^ while maintaining a steady-state level of MnSOD protein.

To further investigate the effects of 4OH-PCB11 on mitochondria, we assessed cellular respiration with the XF96 Extracellular Flux Analyzer. Because 4OH-PCB11 decreased the growth rate of SIRT3-KO MEFs, we also expected the OCR to decrease. On the contrary, the basal and ATP-linked OCRs increased in PCB-treated SIRT3-KO cells. Moreover, the reserve respiratory capacity (i.e., the difference between maximal and basal OCR) decreased drastically in SIRT3-KO cells treated with 4OH-PCB11. This observation strongly suggests that 4OH-PCB11 treatment in the absence of SIRT3 produces cellular stress, perhaps by damaging mitochondrial DNA or electron transport chain (ETC) complexes, requiring additional energy to respond to inefficiency in oxidative phosphorylation. To provide for this excess energy demand, it is likely that SIRT3-KO MEFs increase their OCR by exhausting their reserve respiratory capacity. SIRT3 targets numerous mitochondrial ETC complexes [45] to regulate the activity of the complexes and the rates of electron flow. Thus, the damaging effects of stressors such as 4OH-PCB11 could be exacerbated in the absence of functional SIRT3.

Another mechanism that mammalian cells could use to respond to exogenous stressors like PCBs is metabolic reprogramming. For example, when energy metabolism is perturbed by genotoxic stress, fatty acid oxidation (FAO) can be initiated as an adaptive response [46]. Because SIRT3-KO cells demonstrated decreased growth and increased OCRs (at the expense of their reserve respiratory capacity), we explored whether 4OH-PCB11 treatment also altered FAO-driven respiration or the expression of genes involved in regulating fatty acid metabolism. We found that basal levels of endogenous FAO-associated oxygen consumption decreased significantly in SIRT3-KO MEFs compared to WT, which is consistent with information in the literature [32,47,48] demonstrating that SIRT3 targets FAO enzymes for deacetylation. Interestingly, when SIRT3-KO and WT cells were treated with 3 μM 4OH-PCB11 for 24 h, the FAO-associated OCR decreased in WT MEFs; however, SIRT3-KO MEFs demonstrated an opposite response to 4OH-PCB11 treatment by doubling their endogenous FAO-driven OCR. This suggests that there are additional mechanisms by which PCBs can alter substrate utilization in the absence of SIRT3. We also cannot disregard the possibility that mitochondria become uncoupled in these cells with limited substrate media. FAO-associated OCR measurements performed with exogenous substrates (BSA-conjugated palmitate) revealed similar results for vehicle-treated WT and SIRT3-KO MEFs, where KO cells had a significantly lower OCR than WT cells. However, when both WT and SIRT3-KO MEFs received 3 μM 4OH-PCB11 for 24 h, the exogenous FAO-associated respiration decreased (more drastically in SIRT3-KO cells), implying that 4OH-PCB11 could affect long-chain fatty-acid transport into the mitochondria. It should also be noted that some of our results contradicted previously published data [49]. The differences in culturing conditions in each study, specifically oxygen levels, must be taken into consideration while interpreting the results in these studies.

Because our FAO respiration studies (especially exogenous FAO results) were not conclusive about the effects of 4OH-PCB11 on metabolic reprogramming in the presence and absence of SIRT3 function, we next assessed the expression of genes involved in regulating fatty acid metabolism. Treatment with 3 μM 4OH-PCB11 for 24 h significantly increased the expression of the following genes in SIRT3-KO cells: Acsbg2, Acsm2, Acsl1, Acot12, Hmgs2, Oxct2a, Gk2, Lpl, Slc27a5, and Fabp1. Fatty acids play many important roles, especially as an energy source and as building blocks for triacylglycerols, cholesteryl esters, phospholipids, and the signaling molecule diacylglycerol. These processes start with a common “activation” step in which fatty acids are converted to fatty acyl CoA by acyl-coenzyme A synthetases [50]. When treated with 4OH-PCB11, SIRT3-KO cells displayed significant increases in the expression of three acyl-CoA synthesases: Acsbg2, Acsm2, and Acsl1. Long-chain fatty acid-CoA ligase (Acsbg2) facilitates the activation of long-chain fatty acids for both the synthesis and degradation of cellular lipids. Mitochondrial acyl-CoA synthetase (Acsm2) and long-chain fatty-acid CA ligase 1 (Acsl1) activate long-chain and medium-chain fatty acids, respectively. We also observed an increase in the expression of acyl-coenzyme A thioesterase 12 (Acot12), which is responsible for hydrolyzing acetyl CoA to acetate and CoA; it plays a key role in the synthesis of cytosolic acetyl-CoA, which in turn is important for the synthesis of fatty acids and cholesterol [51,52,53].

Two genes involved in ketone metabolism, mitochondrial succinyl-CoA:3-ketoacid coenzyme A transferase 2A (Oxct2a) and hydroxymethylglutaryl-CoA (HMG-CoA) synthase (Hmgs2), were both upregulated in SIRT3-KO MEFs following treatment with 4OH-PCB11. Oxct2a, involved primarily in spermatogenesis, catabolizes ketone bodies and transfers CoA moieties from succinate to acetoacetate [54], while Hmgs2 condenses acetyl-CoA and acetoacetyl-CoA during cholesterol synthesis and ketogenesis [55]. Additionally, 4OH-PCB11 increased the expression of genes related to triacyglocerol metabolism, lipoprotein lipase (Lpl) and glycerol kinase (Gk2), which regulate the hydrolysis of triglycerides making up circulating chylomicrons and the degradation of glycerol to glycerol phosphate. The upregulation of these genes is associated with metabolic diseases such as diabetes [56,57]. The last two genes that were upregulated by 4OH-PCB11 were bile acyl-CoA synthetase (Slc27a5) and fatty-acid binding protein (Fabp1), which bind and transport long-chain fatty acids into cells. Slc27a5, a member of solute carrier family 27, can also mediate the entry of secondary bile acids into the liver. On the other hand, Fabp1 can bind to cholesterol, bilirubin, fatty acids, and their coenzyme A derivatives [58,59]. In light of several reports demonstrating elevated serum triglycerides and cholesterol levels in PCB exposed populations [60,61,62,63,64] our results could propose a molecular link between PCB induced mitochondrial dysfunction and their metabolic consequences in humans, including increased incidences of diabetes, cardiovascular disease, and hypertension.

Finally, we investigated whether there was a causative relationship between the SIRT3-mediated increase in MnSOD activity in response to 4OH-PCB11 and PCB-induced effects on FAO-related gene expression and mitochondrial function. When we overexpressed MnSOD in SIRT3-KO MEFs with and without 4OH-PCB11, we found no significant differences in the expression of the following six genes: Acot12, Acsl1, Gk2, Hmgs2, Lpl, and Oxct2a, suggesting increased MnSOD activity was able to revert the increased gene expression of genes associated with fatty acid biosynthesis, metabolism, and transport. Overall, our results demonstrate that MnSOD activity plays a significant role in regulating mitochondrial function as well as the expression of genes involved in regulating fatty acid biosynthesis, metabolism, and transport during exposure to 4OH-PCB11.

## 5. Conclusions

Collectively our results suggest that MnSOD is not the only effector in this process, as SIRT3 targets many mitochondrial proteins for deacetylation that could have direct effects on fatty-acid oxidation in the presence of PCBs. It is important to note that short and long-term effects of 4OH-PCB11 on the regulation of genes and proteins associated with FAO must be cautiously considered. Increasing oxygen consumption through electron transport chain and fatty acid oxidation might improve cell survival in short-term, however long-term accumulation of the FAO substrates and intermediates could enhance the electron leak through uncoupled mitochondria during extended exposures to PCBs. Regulation of different mechanisms in addition to SIRT3 and MnSOD activation could also present a different framework for protection against PCB exposures when the exposure times are longer than 24 h.

It will be worthwhile to perform additional in vitro and in vivo studies of SIRT3 to explore the effects of short- and long-term exposure to 4OH-PCB11 and other nonlegacy PCBs on human health. It is critical that we advance our understanding of the mechanisms by which environmental toxicants affect human health with the goal of developing effective countermeasures.

## Supplementary Materials

The following are available online at http://www.mdpi.com/2076-3921/7/9/121/s1, Table S1: Mouse Fatty Acid Metabolism RT^²^ Profiler™ PCR Array, Table S2: Primers used in single gene qRT-PCR. Figure S1: The gas chromatogram and mass spectrum of 4OH-PCB11.
