# Supplementary material for: PCB11 Metabolite, 3,3’-Dichlorobiphenyl-4-ol, Exposure Alters the Expression of Genes Governing Fatty Acid Metabolism in the Absence of Functional Sirtuin 3: Examining the Contribution of MnSOD
Source: Antioxidants (Basel). 2018 Sep 15;7(9):121. doi: 10.3390/antiox7090121 (PMC6162768; doi:10.3390/antiox7090121)
Supplement: Supplementary file 1 [file antioxidants-07-00121-s001.docx]

**Supplementary Material:**

**Table S1.** Mouse Fatty Acid Metabolism RT² Profiler™ PCR Array.

| **PCR Array Gene List** |
| --- |
| **Fatty Acid Metabolism**  Acetyl-CoA Transferases: *Acaa1a, Acaa2, Acat1, Acat2.*  Acyl-CoA Dehydrogenases: *Acad9, Acad10, Acad11, Acadl, Acadm, Acads, Acadsb, Acadvl, Ehhadh, Gcdh.*  Acyl-CoA Oxidases: *Acox1, Acox2, Acox3.*  Acyl-CoA Synthetases: *Acsbg1, Acsbg2, Acsl1, Acsl3, Acsl4, Acsl5, Acsl6, Acsm2, Acsm3, Acsm4, Acsm5.*  Acyl-CoA Thioesterases: *Acot2, Acot3, Acot6, Acot7, Acot8, Acot9, Acot12.*  Carnitine Transferases: *Cpt1a, Cpt1b, Cpt1c, Cpt2, Crat, Crot.*  Other Fatty Acid Metabolism Genes: *Aldh2, Decr1, Decr2, Echs1, Hadha, Mcee, Mut, Eci2 (Acbd2, Peci), Pecr, Ppa1.*  **Fatty Acid Transport**  *Cpt1a, Cpt1b, Cpt1c, Cpt2, Crat, Crot, Fabp1, Fabp2, Fabp3, Fabp4, Fabp5, Fabp6, Slc27a1, Slc27a2, Slc27a3, Slc27a4,*  *Slc27a5, Slc27a6.*  **Fatty Acid Biosynthesis Regulation**  *Prkaa1 (Ampk), Prkaa2, Prkab1, Prkab2, Prkaca, Prkacb, Prkag1, Prkag2, Prkag3.*  **Ketogenesis & Ketone Body Metabolism**  *Bdh1, Bdh2, Hmgcl, Hmgcs1, Hmgcs2, Oxct2a.*  **Triacylglycerol Metabolism**  *Gk2, Gpd1, Gpd2, Gk, Lipe, Lpl.* |

**Table S2.** Primers used in single gene qRT-PCR.

| **PRIMER** | **SEQUENCE** |
| --- | --- |
| Acsm2 fwd | ACT AAT ACC CAT ACA GTG GGG C |
| Acsm2 rev | CTG AAG ATC TCT TGC CAG CCT TC |
| Acsl1 fwd | ATC TGG TGG AAC GAG GCA AG |
| Acsl1 rev | TCC TTT GGG GTT GCC TGT AG |
| Acsbg2 fwd | AGT CTC TGA TCA AGC TCG GC |
| Acsbg2 rev | GAT ACT CAC GTT GGC CTG CT |
| Acot12 fwd | CCG TGG CAC TAA GGT CAG TT |
| Acot12 rev | ACG TTA CGG TGC ACG AAT TG |
| Fabp1 fwd | GTG ACT GAA CTC AAT GGA GAC AC |
| Fabp1 rev | GTA GAC AAT GTC GCC CAA TGT CA |
| Gk2 fwd | GCC TCG AAG CAA ACC TCT G |
| Gk2 rev | TGT GTC AGT TCC ACC TGA TGA |
| Hmgcs2 fwd | GAA GAG AGC GAT GCA GGA AAC |
| Hmgcs2 rev | GTC CAC ATA TTG GGC TGG AAA |
| Oxct2a fwd | GGT AGA TGA CAT CAA GGC CAC |
| Oxct2a rev | TGC TAA GGA TGT CCA GTT GTC |
| SLC27A5 fwd | TCC TGC GGT ACT TGT GTA AC |
| SLC27A5 rev | TCG AAC TGC ACC AGC TCA AAG |
| Lpl fwd | GCG TAG CAG GAA GTC TGA CCA A |
| Lpl rev | AGC GTC ATC AGG AGA AAG GCG A |


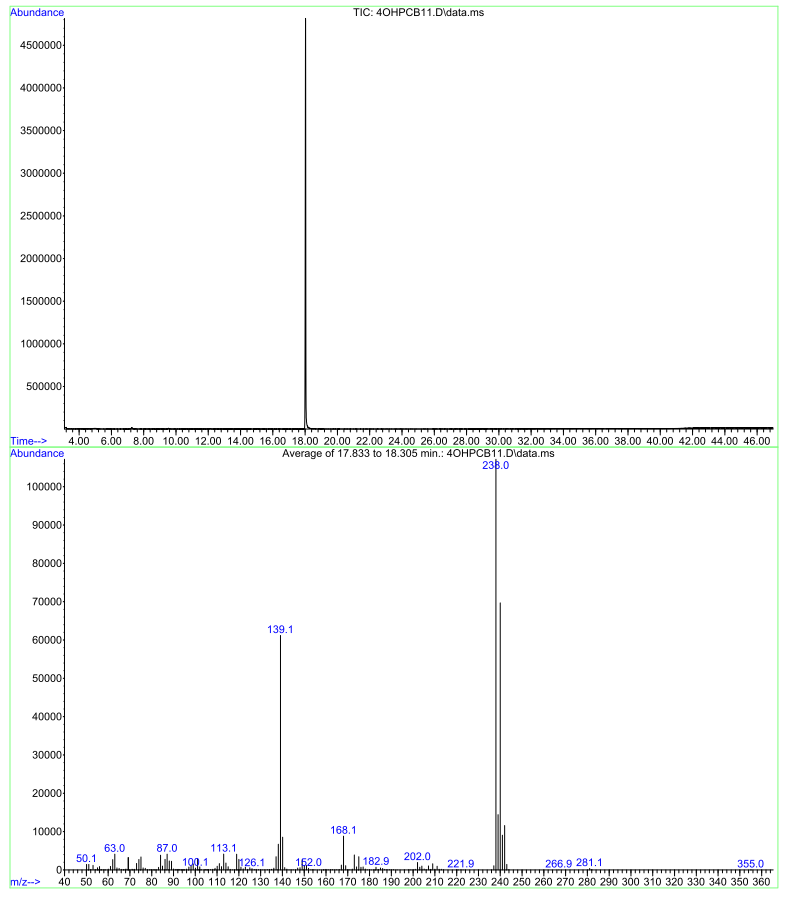


**Figure S1.** The gas chromatogram and mass spectrum of 4OH-PCB11.
